# Detection of Oesophageal Fistula by Radionuclide Salivagram SPECT/CT

**DOI:** 10.3389/fonc.2021.612122

**Published:** 2021-08-24

**Authors:** Yingwei Wang, Chao Wang, Lin Liu, Xinwen Huang, Zhaoyou Guo, Wei Zeng, Rui Sun, Yue Chen

**Affiliations:** ^1^Department of Nuclear Medicine, Affiliated Hospital of Southwest Medical University, Luzhou, China; ^2^Nuclear Medicine and Molecular Imaging Key Laboratory of Sichuan Province, Luzhou, China; ^3^Academician (Expert) Workstation of Sichuan Province, Luzhou, China; ^4^Department of Thoracic Surgery, Affiliated Hospital of Southwest Medical University, Luzhou, China; ^5^Department of Radiology, Affiliated Hospital of Southwest Medical Universitye, Luzhou, China

**Keywords:** salivagram, oesophageal fistula, SPECT/CT, VFSS, ^99m^Tc-DTPA

## Abstract

**Purpose:**

Videofluoroscopic swallowing study (VFSS) is currently the most widely used clinical examination method for diagnosis of oesophageal fistula, but it has many limitations. Therefore, we evaluated radionuclide salivagram single-photon emission computed tomography (SPECT/CT) as a new method of oesophageal fistula diagnosis.

**Methods:**

We retrospectively evaluated the data of 11 patients (10 men and 1 woman, aged 41 to 70 years, with an average age of 58.6 years) who had clinically suspected oesophageal fistula from January 2019 to October 2020. They underwent radionuclide salivagram SPECT/CT and VFSS examinations, and we analysed and compared the results of the two examinations.

**Results:**

A total of 11 patients were included in this study. Ten underwent both salivagram and VFSS examinations. One patient was unable to swallow the contrast agent; therefore, only salivagram was performed, and we excluded this patient from the VFSS analysis. A total of 11 patients underwent salivagram examinations, of which 6 were positive and 5 were negative. A total of 10 patients were tested by VFSS, of which 6 results were positive and 4 were negative.

**Conclusion:**

Radionuclide salivagram SPECT/CT and VFSS are complementary, which can greatly improve the clinical diagnosis and prognosis of oesophageal fistula. When the patient cannot perform the VFSS, or the clinical symptoms are inconsistent with the VFSS imaging findings, the salivagram is an ideal test method.

## Introduction

Oesophageal fistula is a severe complication of oesophageal or lung cancer treatment, especially after radiation therapy (1–3). It has been reported that foreign bodies in the oesophagus can also cause oesophageal fistulas (4). There are many types of oesophageal fistulas, including pleural, aortic, and atrial. Among them, oesophageal-mediastinal fistula and tracheo-oesophageal fistula are the most common, with the latter more common than the former (5).

At present, clinical diagnosis of oesophageal fistula is mainly based on clinical symptoms and imaging, the latter including multidetector computed tomography (CT) and videofluoroscopic swallowing study (VFSS) (6, 7). Many diseases can have the same clinical symptoms. It is difficult to establish the diagnosis or the location and size of the fistula by clinical symptoms alone. CT lacks sensitivity to detect a small fistula, and VFSS is the most commonly used examination. However, clinical application of VFSS has limitations. For example, the procedure is difficult if 1) the patient cannot cooperate with bodily position changes during the examination; 2) the patient has a large tracheo-oesophageal fistula or more severe complications; 3) a gastric tube is present; or 4) the patient has severe dysphagia. Patients in these circumstances cannot achieve the expected diagnostic results if tested by VFSS. Therefore, we sought a diagnostic method without the above limitations.

The advantages of radionuclide inspection are safety, convenience, high patient tolerance, and no special requirement for patient location during the procedure. Also, a combination with SPECT/CT can more accurately determine the location, size, and tissue surrounding the lesion. In this study, the oesophageal fistula was diagnosed using radionuclide salivagram and the results were compared with those of VFSS.

## Materials and Methods

We retrospectively examined the data of 11 patients who underwent radionuclide salivagram SPECT/CT and VFSS in our hospital from January 2019 to October 2020 (**Table 1**). Two associate chief physicians read the films independently to minimise the impact of subjective factors. The final diagnostic result was established by consensus between the two physicians. The patients had one or more of the following medical conditions: 1) oesophageal tumours; 2) history of radiation therapy for oesophageal tumours; 3) history of incarcerated oesophagus with sharp foreign bodies; 4) prominent cough and fever; and 5) CT examination for suspected oesophageal fistula. This study involved no identifying patient characteristics and was approved by the hospital ethics committee. The requirement for informed consent was waived due to the study’s retrospective design.

**Table 1:** Basic patient information and results of salivagram and VFSS.

Patient	Age	Sex	Cause of disease	Salivagram	VFSS	Final diagnosis
1	67	M	Surgery for Carcinoma oesophagus	+	+	Oesophageal fistula
2	55	M	Surgery for Carcinoma oesophagus	+	+	Oesophageal fistula
3	67	M	Surgery for Carcinoma oesophagus	+	+	Oesophageal fistula
4	41	M	Surgery for Carcinoma oesophagus	–	–	Without oesophageal fistula
5	54	M	Oesophageal tumour	+	+	Oesophageal fistula
6	65	M	Oesophageal tumour	+	+	Oesophageal fistula
7	55	M	Oesophageal tumour	–	–	Without oesophageal fistula
8	70	M	Radiotherapy for oesophageal carcinoma	–	–	Without oesophageal fistula
9	49	M	Radiotherapy for oesophageal carcinoma	+	N	Oesophageal fistula
10	66	F	Oesophageal foreign body	–	–	Without oesophageal fistula
11	56	M	Surgery for Carcinoma oesophagus	–	+	Without oesophageal fistula

+, Positive; −, Negative; N, No examination.

### Salivagram

We selected technetium-99^m^ diethylenetriaminepentaacetic acid (^99m^Tc-DTPA) as a radionuclide imaging agent. Before the examination, the patient needed no special preparation. ^99m^Tc-DTPA with a dose of 1 mCi and a volume of about 1-2 mL was dropped onto the patient’s tongue twice with a syringe. The time interval between the two applications was 30 min. After the administration, the patient was instructed to move around and try to swallow as much as possible. A Siemens Symbia T16 SPECT/CT (Siemens, Munich, Germany) was used for static and forward acquisition at 30 min, 60 min, and 120 min after the last dose. The peak energy was 140 Kev, the window width was 20%, the matrix was 128 × 128, the magnification was 1.0, the acquisition count was 500 K, and the patient was supine. In advanced position, the visual field included the oral cavity, oesophagus, bilateral lung fields, and gastric cavity.

If a patient’s oesophageal fistula is suspected to be unilateral, the patient can be ordered to lie on the affected side to increase the positivity rate. During the acquisition process, if the imaging agent is observed outside the digestive tract, local SPECT/CT tomosynthesis imaging can be performed immediately, and the scanning range is consistent with that of the static acquisition: voltage 120 Kv, current 100 mAs, layer thickness 5 mm, pitch 0.8 mm, rotation time 1.0 s, reconstruction using FBP algorithm, convolution kernel B40s, reconstruction of soft tissue window, layer thickness 3.0 mm, layer spacing 3.0 mm. Finally, Siemens post-processing software Syngo MI VA70A was used for image fusion analysis.

### Diagnosis

A positive diagnosis was determined when any images in the acquisition showed imaging agent concentrated outside the digestive tract. A negative diagnosis was determined when during the entire examination, only normal digestive tract images were found.

For patients with obvious clinical symptoms and history of oesophageal surgery or radiotherapy, but no abnormalities on imaging, we can repeat the administration of 1 mCi ^99m^Tc-DTPA once and repeat imaging after 2-4 hours, and routinely perform tomographic fusion imaging. We analysed the positive cases and found that the highest positivity rate was between 60 and 120 min after the second dose of imaging agent.

### Videofluoroscopic Swallowing Study

Patients need no special preparations (although gastric tubes must be removed). The procedure was performed according to the routine VFSS protocol, and we observed whether there was leakage of contrast medium outside the gastrointestinal tract under fluoroscopy.

## Results

Salivagram: Among the 11 patients analysed (10 men and 1 woman, aged 41 to 70 years, with mean age 58.6 years), 6 were positive (6 men; mean age, 59.5 years), and 5 were negative (4 men and 1 woman; mean age, 57.6 years).

VFSS: Ten of the 11 patients analysed (9 men, 1 woman; aged 41 to 70 years, with mean age 59.4 years) completed VFSS; 6 were positive (6 men; mean age, 60.6 years) and 4 were negative (3 men and 1 woman; mean age, 58 years). In one patient with a positive diagnosis, tomographic fusion images confirmed that it was a false positive.

**Figure 1:**
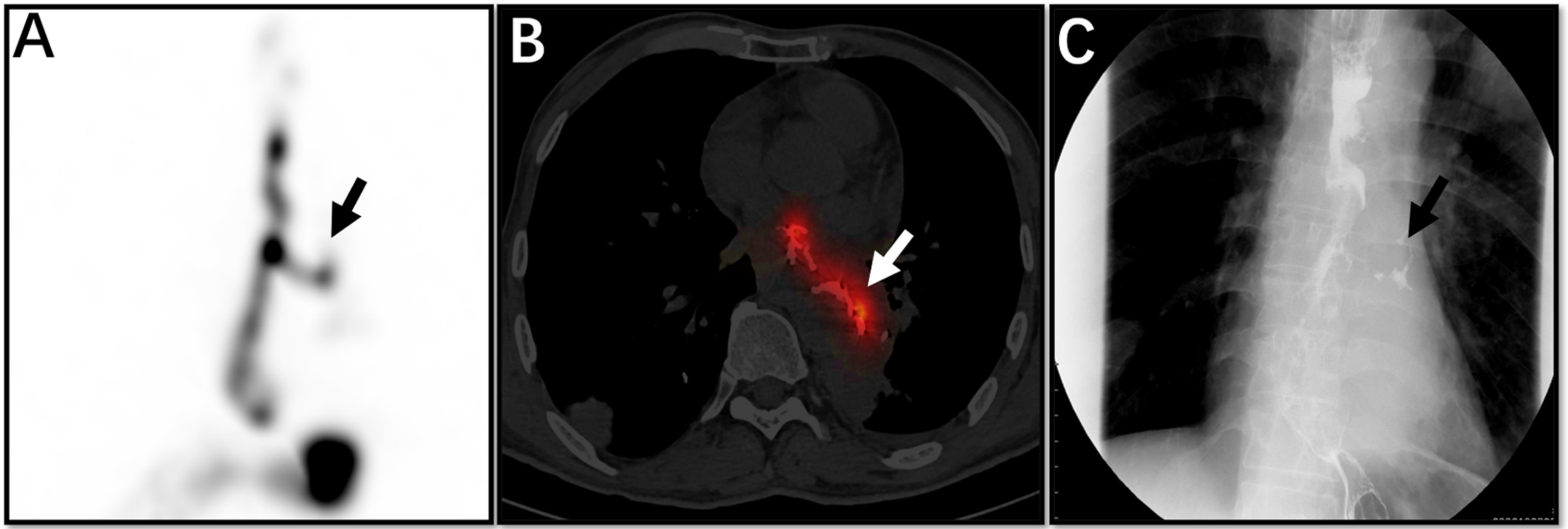
The patient was a 53-year-old man with oesophageal cancer and recurrent cough. Whole SPECT imaging **(A)**, flued SPECT/CT imaging **(B)**, and VFSS imaging **(C)** are demonstrated. These strip imaging agents are sites of oesophageal fistula (arrows). This patient underwent a barium swallow of the upper gastrointestinal tract before salivagram, so the high-density substance in the image is barium **(B)**.

**Figure 2:**
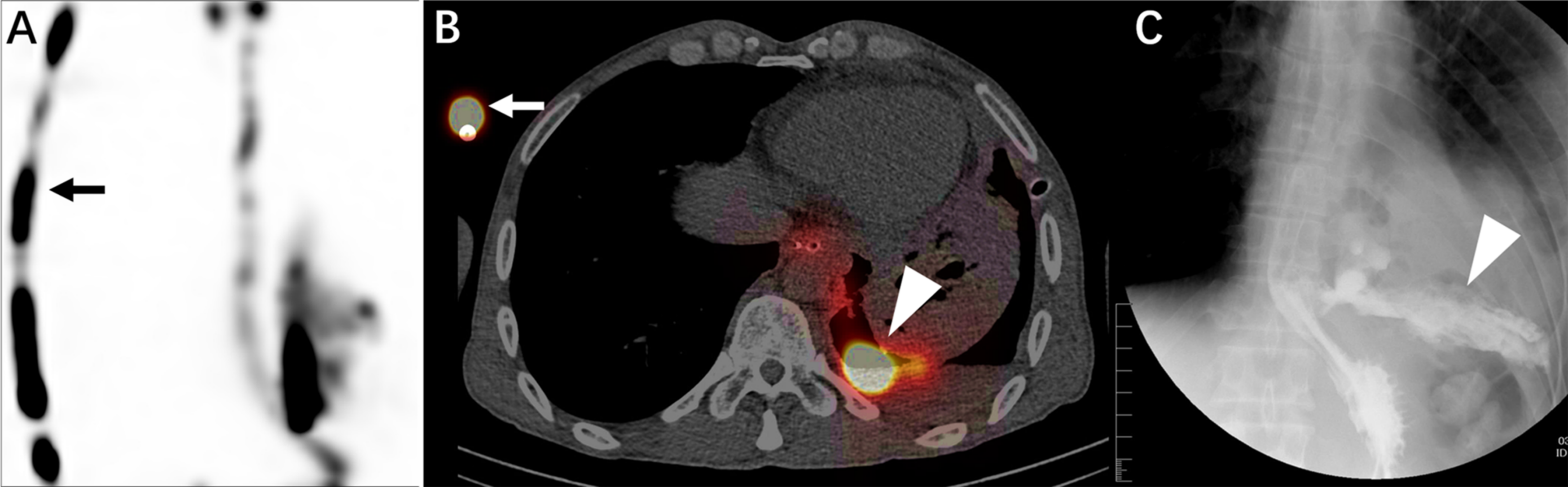
This is a 48-year-old male patient with an oesophageal tumour and requirement for a gastric tube due to inability to eat. The patient underwent salivagram without gastric tube removal. Whole SPECT imaging **(A)**, flued SPECT/CT imaging **(B)**, and VFSS imaging **(C)** are demonstrated. The gastric tube (**A, B**, straight arrow) is visible. An abnormal concentration of imaging agent is present in the left thoracic cavity (**B, C**, triangular arrow).

**Figure 3:**
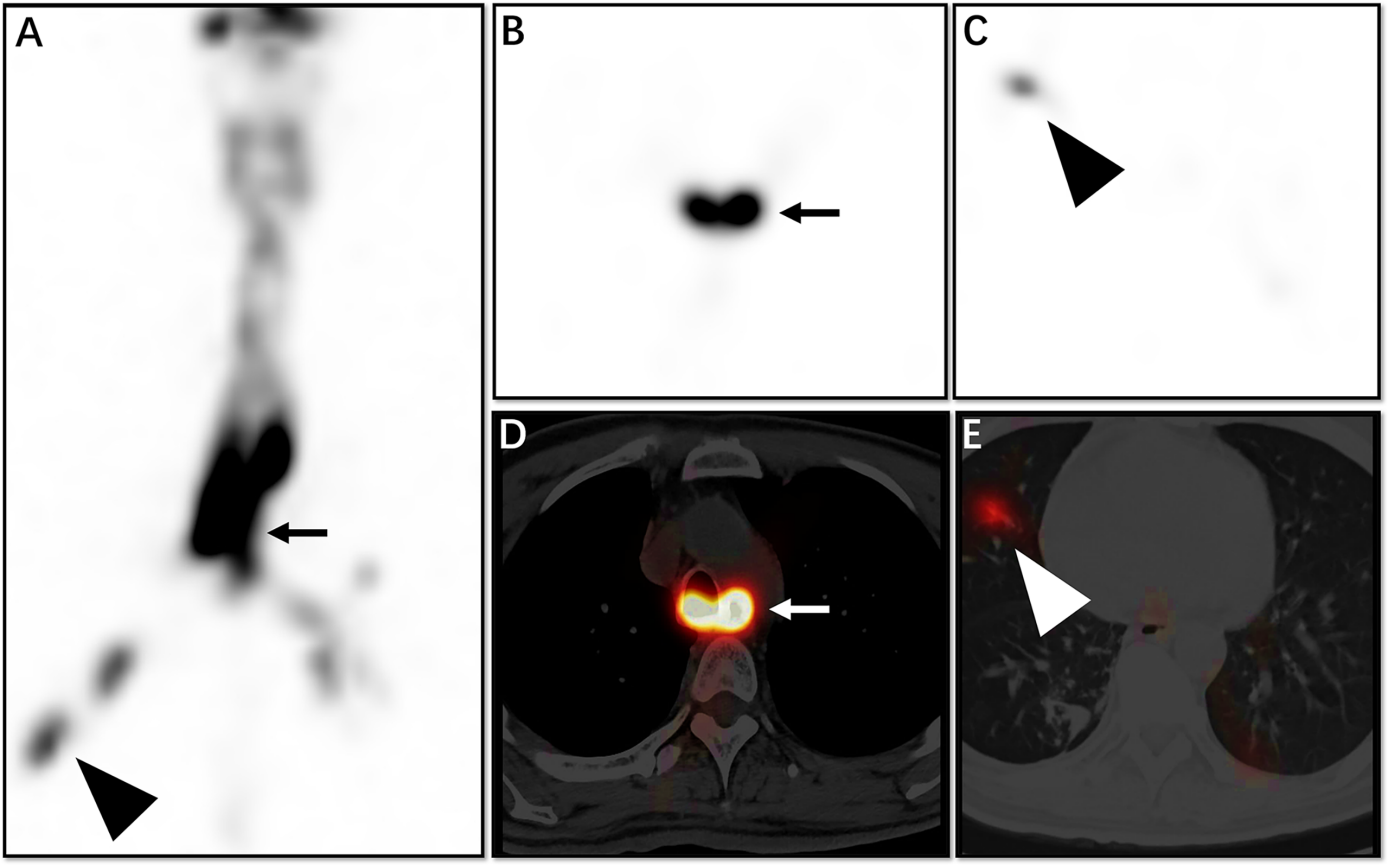
This 43-year-old man had inability to swallow and recurrent coughing. Whole SPECT imaging **(A)**, tomography SPECT imaging **(B, C)**, and flued SPECT/CT imaging **(D, E)** are demonstrated. We see from the image that the opening of the oesophageal fistula is located at the plane of the main bronchial bifurcation (**A, B, D**, straight arrow), and imaging agent is also seen at the distal bronchus (**A, C, E**, triangular arrow).

**Figure 4:**
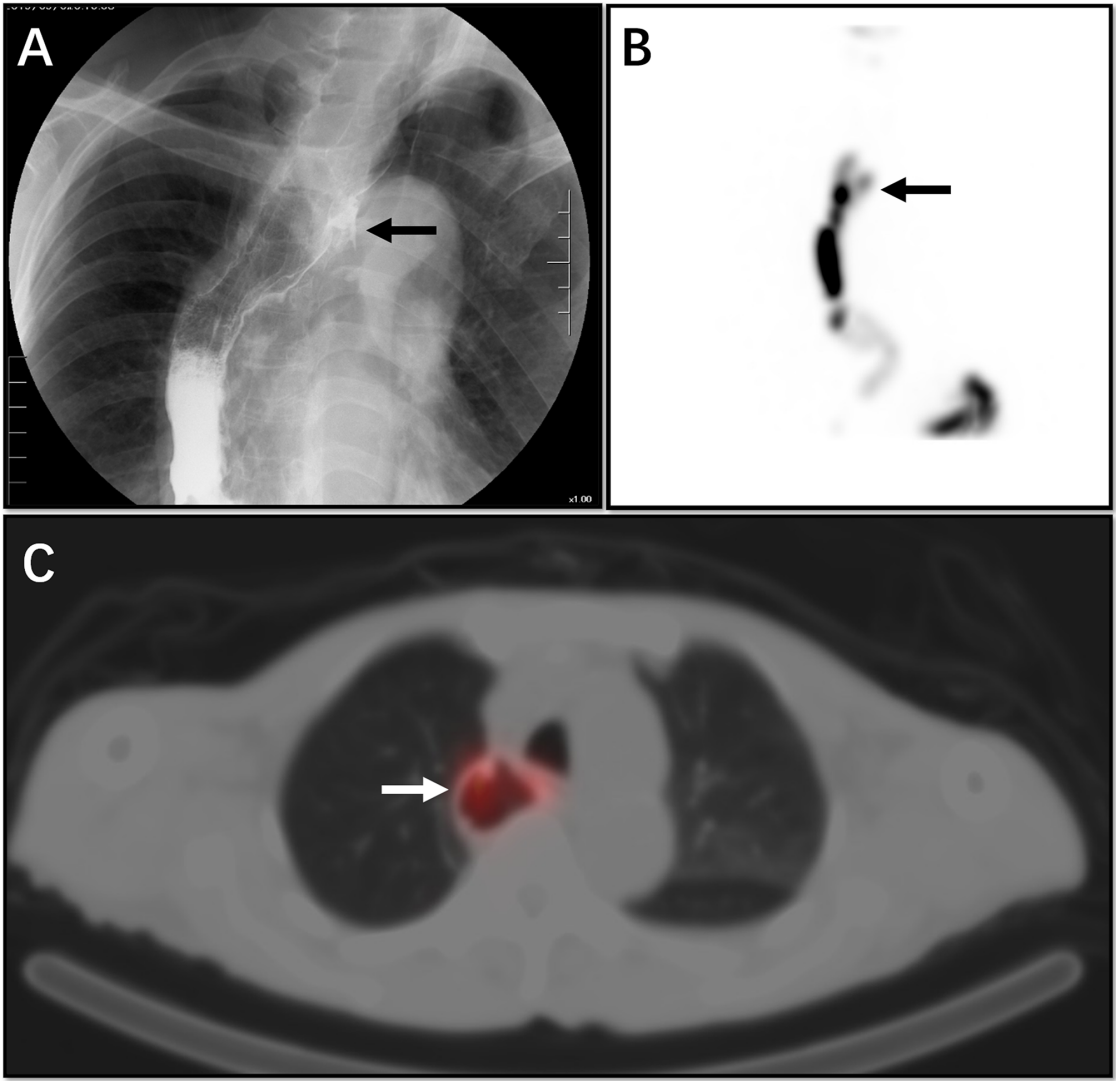
This 53-year-old male patient had a partial oesophageal resection due to an oesophageal tumour. The patient had a VFSS examination, which suggested an upper oesophageal fistula (**A**, arrow). The patient also underwent salivagram. From the MIP image, we see a small concentration of imaging agent outside the lumen of the upper oesophagus (**B**, arrow). On tomographic fusion imaging, we see that the abnormality represents a diverticulum at the anastomosis of the oesophagus (**C**, arrow) rather than an oesophageal fistula.

We compared the results of the two tests in the patient group, each test having its own advantages and disadvantages. Among the 11 patients who underwent salivagram, there were 6 positive diagnoses and 5 negative diagnoses. The demonstrated image was acquired after 2 hours of administration of the radiopharmaceutical. Comparing the images of all patients, we found that images taken at 60-120 min after dosing had the highest positivity rate (**Figure 1**). Among the patients, one (patient 9) had severe clinical symptoms (wheezing) and was unable to swallow the contrast agent for VFSS testing. Two patients had indwelling gastric tubes, and salivagrams were performed without removing the tubes (**Figure 2**). Therefore, only the salivagram was performed, which illustrated the advantage of salivagram (**Table 1** and **Figure 3**). Patient 11 (**Table 1**) was examined in post-oesophageal tumour surgery. VFSS examination suggested that an upper oesophageal fistula had formed. However, in the tomographic fusion images, we recognised that the fistula suggested by VFSS was a diverticulum at the oesophageal anastomosis (**Figure 4**). Through tomographic fusion imaging, we were able to diagnose the lesion more accurately.

## Discussion

One of our patients was examined after surgery for oesophageal cancer. The physician sought to evaluate the patient’s postoperative recovery. He was examined by salivagram in our department. Two hours after the administration of the imaging agent, we found a limited outward projection of the middle and lower oesophagus. The agent concentration was focal, but it was uncertain whether it represented an artefact. Therefore, we asked the patient to undergo another examination after 2 hours. We were surprised to find that the previously visualised lesion had disappeared; therefore, we considered it to have been an artefact. We found no oesophageal fistula in this patient. We followed up the patients and the final clinical diagnosis was consistent with our conclusions. We believe that there are many causes for this performance: 1) various factors, such as reduced oesophageal peristalsis frequency; 2) decreased saliva production; 3) oesophageal diverticulum. When the presence of a lesion is unclear in early imaging, delayed imaging can be performed to improve the accuracy of the diagnosis (8).

With extension of the imaging time, improvement in the accuracy of disease diagnosis has been confirmed in many reports (9). Another patient had a malignant oesophageal tumour and a partial oesophageal resection. The patient was examined by VFSS and found to have an apparent small fistula in the upper oesophagus. To further evaluate the apparent fistula, the patient was examined by salivagram. On the maximum intensity projection (MIP) image, we saw the abnormal concentration of the imaging agent outside the lumen of the upper oesophagus, but our tomographic fusion showed that this was not a fistula. Rather, it was a small diverticulum of the gastro-oesophageal anastomosis.

In our study, we found that the optimal time for image acquisition after administration of imaging agent was 60-120 min. In some patients, although an oesophageal fistula is highly suspected clinically, imaging results can be negative. With salivagram, we can use a consistent method, one repeat dose of imaging agent, and acquire images after 2-4 h. If necessary, we can also perform tomographic fusion imaging to minimise misdiagnosis.

Bivins et al. proved in their 1977 study that radionuclide imaging has potential value in the diagnosis of oesophageal fistula (10). Radionuclide imaging has long been used clinically, and its effectiveness was proven in 1980 (11). Dunn et al. proved in a 1983 study that radionuclide imaging has further research value in the diagnosis of an oesophageal fistula (12). Since then, many studies have shown that this method is highly sensitive for detecting aspiration of saliva (11, 13). Our research is also based on the above report. However, so far, most salivagram examinations have been performed for children and rarely for adults (13–18).

Previous studies have shown that salivagram is often used in the diagnosis and treatment of congenital oesophageal atresia, tracheo-oesophageal fistula, and aspiration pneumonia in children (19–22). From these reports, we can also infer that salivagram is a safer method of inspection. Reports regarding the diagnosis of an oesophageal fistula using salivagram are scarce. Judging from the current equipment in nuclear medicine departments, the popularity of SPECT is high, and almost all nuclear medicine departments perform it. ^99m^Tc-DTPA is a commonly used radiopharmaceutical in nuclear medicine. This combination provides good equipment and imaging preparation to improve the popularity of salivagram.

As mentioned earlier, many diseases can lead to oesophageal fistulas, which have a high mortality rate, especially those with delayed diagnosis and treatment. Therefore, timely diagnosis and treatment are needed for a better prognosis. Although VFSS is the most common method for diagnosis of oesophageal fistulas, we found that it has many limitations on clinical application. To better solve clinical problems, we conducted this study and found that salivagram can be preferable for diagnosis of oesophageal fistulas in the following situations: 1) the patient is unable to cope with changes in posture during the examination; 2) the contrast agent swallowed by the patient does not achieve the diagnostic effect; 3) the clinical diagnosis is inconclusive with VFSS; 4) the patient has tracheo-oesophageal fistula or other serious complications; 5) the physician must evaluate the anatomy around the fistula; 6) the patient has a gastric tube; and 7) the patient has severe dysphagia.

In this study, we found that salivagram is more tolerable for patients, and its rate of diagnosis of oesophageal fistula is also high. It effectively addresses the limitations we have found with VFSS. For patients with a clear history of oesophageal tumours and oesophageal radiotherapy, but no obvious abnormalities on the salivagram, we added SPECT/CT tomography, which can show the anatomical location of the lesion more clearly, minimise false negatives, and provide physicians with more disease information. It can be seen from the previous cases that SPECT/CT tomographic fusion imaging can help us to maximise the sensitivity for diagnosis and minimise false positives. ^99m^Tc-DTPA is a conventional clinical imaging agent, and its safety has been widely recognised (23). We use oral administration, which is also the most natural means of consumption (11).

However, our study also has some limitations. First, because our study sample size is small, the positivity and negativity rates of the two methods are not comparable. This provides scope for further study. Second, compared with VFSS, the examination time for salivagram is longer, especially for patients with very small fistulas, the waiting time for examination is longer, and it may require repeated imaging agent administration and imaging. Our research work also demonstrates new use of a commonly used radiopharmaceutical. We hope to explore the potential value of salivagrams in overcoming the challenges encountered in clinical practice and to therefore reduce patient suffering.

In conclusion, salivagram is a highly tolerable, safe, and reliable examination for patients. Radionuclide salivagram SPECT/CT and VFSS complement each other, which can greatly improve the clinical diagnosis and prognosis of the oesophageal fistula. When the patient cannot perform the VFSS or the clinical symptoms are inconsistent with the imaging findings, radionuclide salivagram SPECT/CT is an ideal testing method.

## Data Availability Statement 

The original contributions presented in the study are included in the article/supplementary material. Further inquiries can be directed to the corresponding author.

## Ethics Statement

The studies involving human participants were reviewed and approved by Affiliated Hospital of Southwest Medical University, Luzhou, Sichuan, China. The patients/participants provided their written informed consent to participate in this study.

## Author Contributions

All authors listed have made a substantial, direct, and intellectual contribution to the work, and approved it for publication.

## Conflict of Interest

The authors declare that the research was conducted in the absence of any commercial or financial relationships that could be construed as a potential conflict of interest.

## Publisher’s Note

All claims expressed in this article are solely those of the authors and do not necessarily represent those of their affiliated organizations, or those of the publisher, the editors and the reviewers. Any product that may be evaluated in this article, or claim that may be made by its manufacturer, is not guaranteed or endorsed by the publisher.
